# The reliability and predictive validity of a sixth-semester OSPE in conservative dentistry regarding performance on the state examination

**DOI:** 10.3205/zma001087

**Published:** 2017-02-15

**Authors:** Petkov Petko, Katja Knuth-Herzig, Sebastian Hoefer, Sebastian Stehle, Sonja Scherer, Björn Steffen, Stephan Scherzer, Falk Ochsendorf, Holger Horz, Robert Sader, Susanne Gerhardt-Szép

**Affiliations:** 1 Goethe-Universität, Carolinum Zahnärztliches Universitäts-Institut gGmbH, Poliklinik Zahnerhaltungskunde, Frankfurt am Main, Deutschland; 2 Goethe-Universität, Institut für Psychologie & Interdisziplinäres Kolleg Hochschuldidaktik, Frankfurt am Main, Deutschland; 3 Goethe-Universität, Klinik für Mund-, Kiefer- und Plastische Gesichtschirurgie, Frankfurt am Main, Deutschland; 4 Goethe-Universität, Medizinische Klinik II, Hämatologie und Internistische Onkologie, Frankfurt am Main, Deutschland; 5 Goethe-Universität, Zentrum für Innere Medizin, Frankfurt am Main, Deutschland; 6 Goethe-Universität, Klinik für Dermatologie, Venerologie und Allergologie, Frankfurt am Main, Deutschland

**Keywords:** OSPE, OSCE, predictive validity, reliability, state exams, length of study, clinical competency

## Abstract

**Introduction: **The aim of this study was to ascertain whether the testing format of an OSPE (Objective Structured Practical Examination) in conservative dentistry (sixth semester) predicts the scores on the practical section of the state examination (11^th^ semester) in the same subject. Taking general student profiles into consideration (score on the school-leaving exam [Abitur], score on the preliminary exam in dental medicine [Physikum], length of university study, cohorts, and sex), we also investigated if any correlations or differences exist in regard to the total and partial scores on the OSPE and the corresponding state examination.

**Methods: **Within the scope of this longitudinal retrospective study, exam-specific data spanning 11 semesters for dental students (N=223) in Frankfurt am Main were collected and analyzed. Statistical analysis was carried out by calculating Spearman rank correlations, partial correlations, Pearson’s correlation coefficients, and multiple regressions (SPSS Statistics 21, IBM Corporation, New York).

**Results: **The results show that the OSPE (Cronbach’s α=.87) correlates with level of success on the practical section of the state exam in conservative dentistry (*p*=.01, *r*=.17). Length of university study also emerged to correlate significantly with the state exam score (*p*=.001, *r*=.23). Together, these two variables contribute significantly to predicting the state exam score (*p*=.001, *R^2^*=.076). This was seen extensively among female students. It was also discovered that these female students had higher school-leaving exam scores than male students (*F*=6.09, *p*=.01, *η^2^*=.027), and that a significant correlation between scores on the Physikum (preliminary exam in dental medicine) and OSPE scores existed only for male students (*r*=.17, *p*=.01).

**Conclusion: **This study was able to demonstrate the predictive effect of a clinical OSPE regarding scores achieved on the state exam. Taking the limitations of this study into account, we are able to recommend using the OSPE testing format in the sixth semester during the clinical phase of dental study.

## Zusammenfassung

**Einleitung: **Ziel dieser Studie war es zu evaluieren, ob das Prüfungsformat einer OSPE (Objective Structured Practical Examination) durchgeführt im Fach Zahnerhaltungskunde (6. Fachsemester) den Studienerfolg im praktischen Teil des Staatsexamens (11. Fachsemester) im selben Fach prädiziert. Ferner sollte unter Berücksichtigung allgemeiner Angaben der StudienteilnehmerInnen (Abitursnote, Physikumsnote, Studiendauer, Kohorte und Geschlecht) analysiert werden, ob bezüglich der Gesamt- sowie Teilnoten der OSPE und der adäquaten Staatsexamensprüfung Zusammenhänge oder Unterschiede bestehen.

**Methoden:** Im Rahmen dieser longitudinalen, retrospektiven Studie wurden für einen Zeitraum von 11 Semestern prüfungsbezogene Daten von Studierenden (N=223) des Fachbereichs Zahnmedizin in Frankfurt am Main erhoben und untersucht. Für die statistische Auswertung der Daten wurden Spearman Rangkorrelationen, Partialkorrelationen, Korrelationskoeffizienten nach Pearson, und Multiple Regressionen (SPSS Statistics 21, IBM Corporation, New York) berechnet.

**Ergebnisse: **Die Ergebnisse zeigen, dass OSPE (Cronbachs α=.87) mit dem Erfolg im praktischen Teil des Staatsexamens im Fach Zahnerhaltungskunde korreliert (*p*=.01, *r*=.17). Als eine weitere signifikante Korrelation mit der Examensleistung erwies sich die Dauer des Studiums (*p*=.001, *r*=.23). Gemeinsam leisten diese beiden Variablen einen signifikanten Beitrag zur Vorhersage der Examensnote (*p*=.001, *R^2^*=.076). Das zeigte sich im größeren Umfang bei weiblichen Studierenden. Zudem wurde festgestellt, dass diese bessere Abiturnoten als männliche Studierende aufweisen (*F*=6.09, *p*=.01, *η^2^*=.027) und dass es lediglich bei männlichen Studierenden eine signifikante Korrelation zwischen der Physikumsnote (Zahnärztliche Vorprüfung) und der OSPE-Benotung gab (*r*=.17, *p*=.01).

**Schlussfolgerung: **In der vorliegenden Untersuchung konnte der prädiktive Effekt einer klinischen OSPE auf die Prüfungsleistung im Staatsexamen gezeigt werden. Unter Berücksichtigung der Limitation der Studie empfiehlt sich aus unserer Sicht die Durchführung eines solchen Prüfungsformats im Rahmen des klinischen Studienabschnitts im 6. Semester im Fach Zahnmedizin.

## 1. Introduction and research question

**Figure 1:**
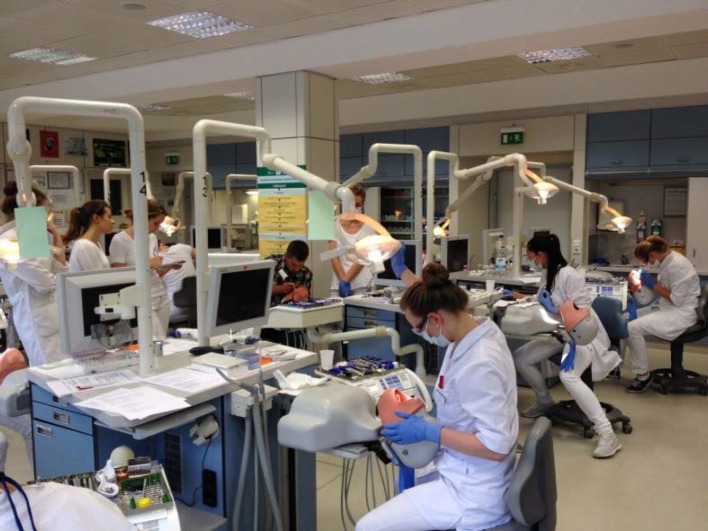
Treatment procedures on phantom patients during the first semester of clinical study

**Figure 2:**
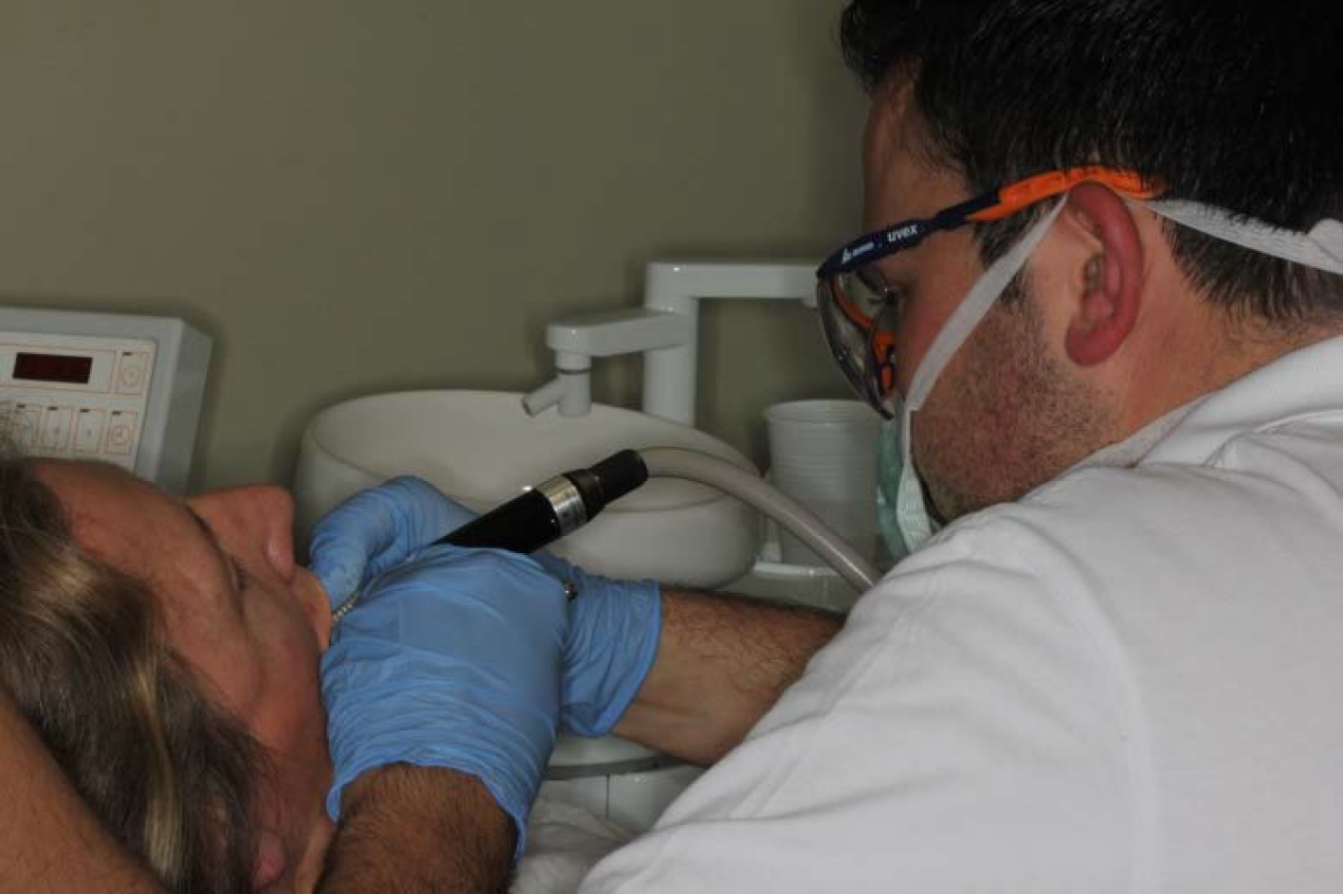
Treating patients during the state exam

Based on the German licensing rules and regulations for dentists (Approbationsordnung) [https://www.gesetze-im-internet.de/_appro_2002/ cited 2015 October 22], dental education consists of a preclinical and clinical study phase, each lasting five semesters. Over the course of this, students acquire both theoretical knowledge and spend a considerable amount of time learning practical skills. During the preclinical phase basic knowledge of natural science and the scientific principles of medicine are taught, while during the later clinical phase, students directly apply the theoretical knowledge acquired in lectures and begin developing professional routines in the first clinical semester, initially using phantom patients (see figure 1 (Fig. 1)) and then as part of multiple courses on treating real patients (see figure 2 (Fig. 2)).

In the first clinical semester the main focus is on conservative dentistry. Students are taught in the simulation lab. To prepare and qualify students for the first course in treating patients, specific procedures are simulated and the required manual skills and techniques of restorative dentistry and endodontics are imparted through systematic training.

Acquisition of dental competencies during education requires close interlinking of teaching and testing, referred to as constructive alignment [1]. Consequently, the analysis of suitable testing formats has been at the center of research in dental education for years now [2]. A broad range of workplace-based assessments are used in dental education, especially in the clinical courses that center on treating patients. Testing formats are used which not only cover students’ factual and practical knowledge, but also their practical skills [3].

Various internationally known testing methods exist to assess students’ practical skills in complex situations, such as the Clinical Evaluation Exercise (CEX) [4], Mini-Clinical Evaluation Exercise (mCEX) [5], Entrustable Professional Activities (EPA) [6], Directly Observed Procedural Skills (DOPS) [6], and portfolios [7], all of which have not found wide use in dental medicine due to various problems such as insufficient content validity, questionable reliability, low acceptance and cumbersome implementation. However, dental education frequently relies on testing formats that reflect the “shows how” level of Miller’s pyramid. These formats assess practical skills and communicative competencies, with specific reference here to the OSCE (Objective Structured Clinical Examination) [8], [9], OSPE (Objective Structured Practical Examination) [10] and the use of trained simulated or standardized patients, for instance as part of an OSCE [11], [12]. In the international literature the terms “OSCE” and “OSPE” are commonly used as synonyms. Since its introduction to the medical curriculum in 1975 [13], the OSCE has been successfully implemented around the world as an assessment tool in a wide variety of subjects [14], including dentistry exams since the 1990s [8], [9], [15]. In addition to its wide acceptance among students, who feel the OSCE is a more just and less stressful testing format than traditional written and oral exams [16], it also strengthens skills in the area of clinical competence [9], [11], [17], fosters both communication skills [9] and learning, and contributes to a more accurate self-assessment by the participants [16], [17], [18].

During an OSCE students go through different stations at which specific practical skills (including individual steps of a treatment) are performed or a doctor-patient conversation is held (simulated patients). A special type of OSCE is the OSPE in which practical skills, knowledge, and interpretation of data are demonstrated in a non-clinical situation (e.g. simulation lab). In contrast to the OSCE, an OSPE provides an opportunity to assess entire work processes (all individual steps and the “finished product,” for instance, a filling or an inlay in the field of conservative dentistry).

**Figure 3:**
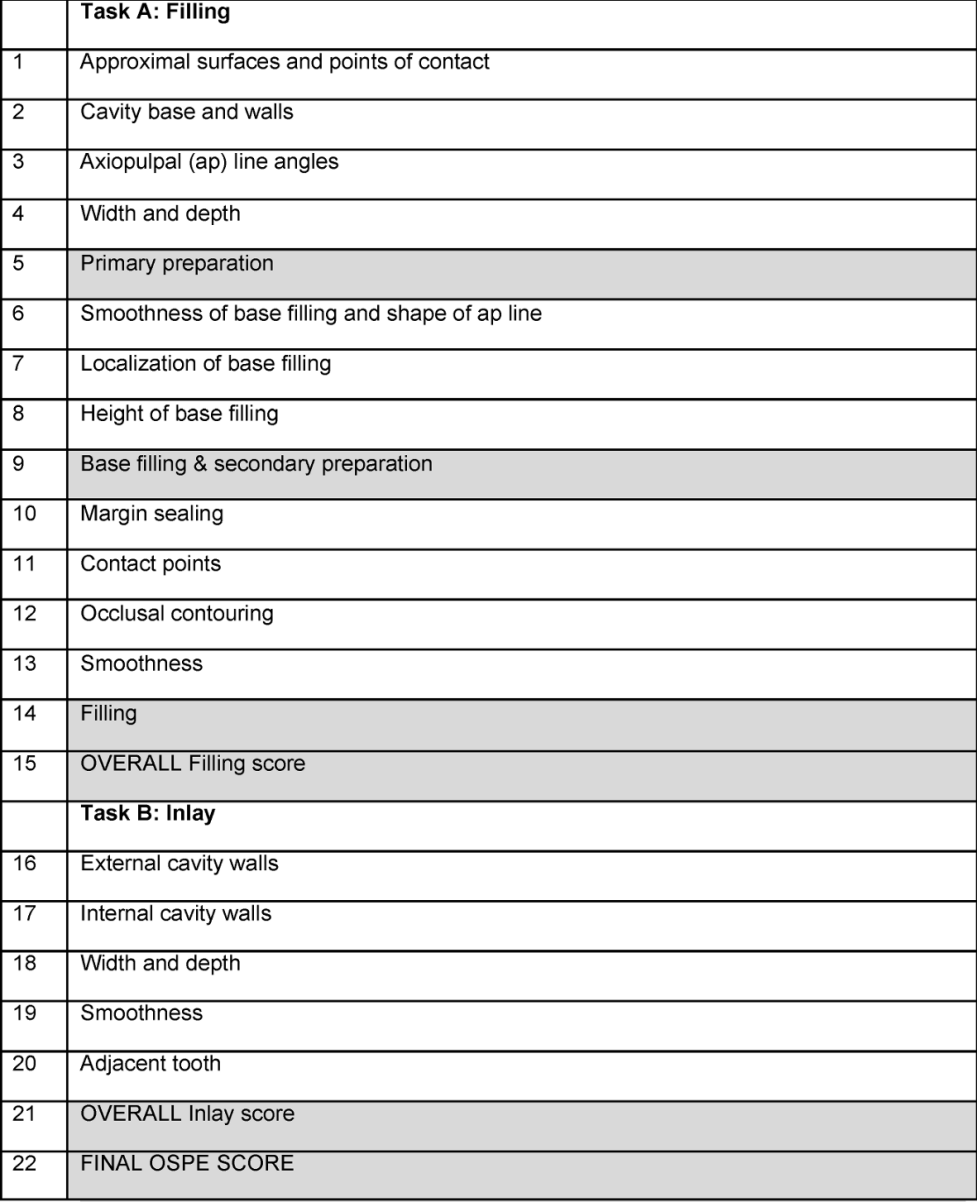
The individual items of the OSPE

The dental program in Frankfurt am Main uses OSCEs (third clinical semester in the subjects conservative dentistry and oral and maxillofacial surgery), OSPEs (at the end of the first clinical semester in conservative dentistry), and simulated patients (in OSCEs). In the final stage of the degree program in dental medicine during the state examination, all students take not only theoretical but also practical tests in which everything must be performed independently on a patient (see figure 3 (Fig. 3)).

Achieving the goal of best preparing students to treat patients requires critical evaluation of the concept underlying the existing rules and regulations in dental education governing the teaching of practical skills and abilities compared to theoretical knowledge of dental medicine. Early detection of deficiencies could be addressed and remedied individually by students and teachers over the course of study leading up to the state examination. However, it would be necessary to show evidence as to whether or not different testing formats correlate with each other, even if they each measure the same competencies (e.g. practical skills and abilities). When doing this, factors such as the sex of a student, length of study, score on the school-leaving exam and first section of the state medical exam should be examined for potential influence and predictive value. Accordingly, the primary aim of this study was to ascertain if, for the subject conservative dentistry, the final score on the OSPE in the sixth semester predicted the later practical exam score. In addition, it was sought to clarify whether or not correlations exist between the partial scores of the OSPE administered in the sixth semester and the corresponding practical scores on the state exam.

## 2. Material and Methods

### 2.1. Study timeline and setting

For this longitudinal retrospective study data was collected for two semesters (first and last clinical semesters). The period investigated was the time between the beginning of the 2009 summer semester and the end of the 2014 summer semester. People for whom information was available regarding all the listed variables were included in the analysis. In addition to dropping out between the OSPE and state exam (n=39, 14.61%), the reasons for exclusion from the calculations included missing data for scores on the Physikum (48 cases), scores on the school-leaving exam (33 cases), sex (six cases), or length of university study (219 cases). Another reason entailed OSPE data for students that was available for current semesters but with which no subsequent state exam scores could be matched at the time of our calculations. For students classified as drop-outs it is not known if they represent authentic instances of quitting university study or if their scores were simply no longer adequately documented.

#### 2.2. Student participants

Participants in the study were students enrolled in dental medicine at the Dental School (Carolinum) of the University of Frankfurt am Main, Germany. This study analyzed the available OSPE scores (retrospectively as of the 2009 summer semester) and the state examination (retrospectively as of the 2011/12 winter semester). Personal information was also evaluated, such as age, sex, school-leaving exam score, preliminary medical examination score, and length of university study, and students were assigned to cohorts. The sample population investigated here was comprised of 223 students, of which 141 were women and 82 men.

#### Academic Setting

##### 2.2.1. Sixth semester

At the end of the first clinical semester (sixth semester of specialized study) oral and practical assessments are given in the subject of conservative dentistry to measure knowledge gain. The semester-end, practical exam at the University of Frankfurt am Main’s Dental School (Carolinum) takes the form of an OSPE, during which individual evaluations are recording using standard checklists. At Frankfurt am Main, three to four examiners are used for OSPEs. The assessment consists of two tasks – a dental filling and an inlay. Both tasks are divided into sub-steps. For the dental filling these are:

1. Primary preparation,
2. Bases/liners and secondary preparation,
3. Filling,
4. Filling overall.

The inlay station is divided into:

1. External cavity walls,
2. Internal cavity walls,
3. Width and depth,
4. Smoothness,
5. Adjacent tooth.

The individual items are presented in figure 3. They are evaluated using the traditional academic grading scale from 1 (very good) to 5 (deficient) and combined into a total score for each station. The overall evaluation of the filling consists of the individual scores for “primary preparation”, “bases/liners and secondary preparation”, and “filling”. At the end of the assessment a final score is calculated for the OSPE by combining the overall scores for the filling and inlay.

##### 2.2.2. Semester of the state examination

As part of the state exam in dental medicine (11^th^ semester of clinical study), the practical assessment in conservative dentistry lasts five days. Examinees are required to independently perform several procedures on patients, among them a root canal, preparation for an inlay and cementing the restoration into place, an anterior tooth filling, a posterior tooth filling, and making a diagnosis in two patients. The testing format used for the state exam covers procedural skills, as does the sixth-semester OSPE, but on the highest level of competency. The two items of overlap between the OSPE administered in the sixth-semester and the state exam – the filling and inlay – are both covered to the same extent. Performance on the state exam is evaluated by two examiners using standardized checklists to assign scores. Filling and inlay stations relevant to this comparative study encompass the following sub-steps. For the filling station:

1. Primary preparation,
2. Rubber dam,
3. Base/liner and secondary preparation,
4. Matrices,
5. Filling before polishing,
6. Filling after polishing,
7. Filling overall.

For the inlay station:

1. Preparation (primary and secondary),
2. Impression,
3. Provisional inlay,
4. Inlay before placement,
5. Inlay after placement,
6. Inlay overall.

The exam checklist contains the same subcategories for the items filling and inlay as the sixth-semester OSPE checklist. The sub-step “rubber dam“ is covered in the OSPE evaluation of bases/liners and secondary preparation; matrices and the steps involving the filling before and after polishing are part of the OSPE section covering the filling. The five sub-steps for the OSPE inlay station are part of “preparation” in the state exam. Steps two through five (impression to inlay after cementing) are not covered by the OSPE because an indirect restoration is not part of that assessment. This would take several days since these fillings would have to be made in a dental lab and thus cannot be realized in a three-hour OSPE. The individual steps in the state exam are also evaluated using the traditional grading scale of 1 (very good) to 5 (deficient).

#### 2.3. Analytical methods

Statistical analysis was performed in cooperation with the Department of Educational Psychology (Frankfurt am Main). Analysis was carried out using SPSS Statistics 21 (IBM Corporation, New York) and calculated Spearman rank correlation, partial correlation, Pearson’s correlation coefficient and (multiple) linear regressions.

## 3. Results

### 3.1. General results

#### 3.1.1. Reliability of the OSPE

Based on all partial scores, The OSPE demonstrates overall a high internal consistency with a Cronbach’s α of .87. The two items for filling (Cronbach’s α=.84) and inlay (Cronbach’s α=.87) also reflect a good internal consistency.

##### 3.1.2. Prediction of state exam scores based on OSPE performance

In the student population investigated here, the final OSPE score correlated to a significantly positive degree with the overall score on the practical section of the state exam (Spearman rank correlation, *r*=.14, *p*=.03). It can be observed that higher OSPE scores go along with higher practical exam scores. Students earned a mean of 3.79 (standard deviation=0.81) on the OSPE and were able to show a mean improvement of approximately two grade levels (M=1.87, SD=.38).

Of the variables investigated, apart from the OSPE score, only the length of study (Spearman rank correlation, *r*=.20, *p*=.003) correlated significantly with the exam score, showing that a higher exam score paired with a shorter length of study, and a longer length of study with a lower exam score.

The calculation of a multiple regression with stepwise addition of the OSPE score and the length of study showed that both contribute significantly to predicting the state exam score (*ß*=.16, *p*=.02, *R^2^*=.08).

##### 3.1.3. Correlation between partial scores on the OSPE and partial scores on the state exam (filling and inlay)

The OSPE scores for filling and inlay were also correlated with the score for the corresponding items on the practical state exam (see table 1 (Tab. 1)). These two items were selected because they allow relatively direct comparison due to extensive overlap of content and suggest a link between scores. It was possible to determine, while examining the cohort as a possible influential factor, that the OSPE and state exam scores for the filling did not correlate significantly with each other (*r*=.12, *p*=.07). The scores assigned for inlays, in contrast, showed such a correlation (*r*=.13, *p*=.05).

Within an exam or assessment, however, these two scores correlated quite highly with each other, meaning that whoever did well (or poorly) on a section of a particular test also did as well (or poorly) on the other section. This is reflected in the good reliability of the OSPE overall assessments (see table 1 (Tab. 1)).

#### 3.2. Gender differences

##### 3.2.1. Scores and length of study

In the sample of 141 female and 82 male students, no statistically significant differences could be determined within the OSPE regarding sex and the partial scores for filling and inlay (indirect restoration) or the final score. Furthermore, in respect to the partial scores and final scores on the state exam there were no gender differences. The same is true for length of study. Gender differences could only be detected for the scores on the school-leaving exam and the Physikum (preliminary exam in dental medicine). The school-leaving exam scores showed that female students (*M*=2.12) had significantly higher scores (*F*=6.09, *p*=.01, *η^2^*=.03) than male students (*M*=2.31). However, no correlations between the school-leaving exam scores could be seen with the OSPE or state exam, and consequently success on the OSPE or practical state exam cannot be predicted by the school-leaving exam score. This could be explained by the use of the school-leaving exam score as a selective criterion when admitting students to the degree program, something that definitively limits its variance in the sample population. In the Physikum it was seen that male students with a mean value of 2.24 performed significantly better (*F*=4.15, *p*=.04, *η^2^*=.02) than female students (*M*=2.40). In male students this score also correlated significantly (*r*=.24, *p*=.03) with the OSPE, with a high OSPE score accompanying a high Physikum score. This was not the case for female students (*r*=.12, *p*=.17). In contrast, no significant correlations between Physikum score and state exam score could be determined.

##### 3.2.2. Using the OSPE to predict the state exam score

If the correlation of the OSPE is considered separately according to sex, it is seen that for female students a higher correlation (*r*=.195, *p*=.02) can be found between the OSPE and the state exam score than is the case for male students (*r*=.137, *p*=.22). Likewise, a high correlation (*r*=.27, *p*=.007) exists between length of study and exam score only for female students if calculations are made separately according to sex. The calculation of a separate multiple regression analysis with stepwise addition of these two variables revealed that only for female students did they contribute significantly to predicting the exam score (*ß*=.18, *p*=.03, *R^2^*=.10). This was lower for male students (*p*=.16, *R^2^*=.05).

## 4. Discussion

Well-known internationally, the testing format applied by the OSCE demonstrates reliability values between Cronbach’s α .40 and .91 [19]. This can be shown for both the purely medical field and dental medicine [10], [18]. The OSPE represents a variation of the OSCE that is focused more on practical skills [10]. Both terms are often used in the literature to mean the same thing [10].

Studies on the predictive validity of the OSCE/OSPE in medicine and dental medicine [8], [15], [17], [18], [20] show that student performance on OSCEs reflects their future clinical competency. Our study confirms a high reliability and a predictive validity of success on the practical state exam in conservative dentistry for a specific format of the OSPE, thus corroborating the studies cited above. In contrast, a study of dental medicine [21] found no significant correlation between OSCE and state exam. A possible reason could be that the overall score for that assessment (both practical and oral assessments) was investigated, while this study concentrates on direct comparison of the practical skills. No other publications are known in which the OSCE/OSPE and state exam have been investigated in terms of scores on identical assessment subsections. Within this context, this study presents a new perspective on this issue. It is surprising that the correlation between OSPE and the practical state exam is much clearer for women, something that raises a series of interesting questions. However, the limitation of this study must be kept in mind. Based on the available data, the potential influence of the task to be performed and rater cannot be analyzed using the methods applied here.

Moreover, it was possible to determine that the scores for inlays correlated significantly with each other for the OSPE and state exam, but this was not the case for fillings.

The reason for this could lie in the different test settings (OSPE versus state exam). However, it remains that whether the assessment is conducted in a clinical setting on phantom patients (OSPE) or on real patients (state exam), the tasks of filling and inlay obviously require a very similar level of learning transfer and manual dexterity within a particular assessment. Within an assessment these two partial scores correlate with each other. When different assessments are compared, the requirements for inlays appear to be more similar than those for the filling. It can be supposed that the ability to visualize spatially, a skill that generally plays an important role in exercising practical skills, exerts an influence across assessment settings.

The general increase in practical competencies from the OSPE (mean score 3.79) to the state exam (mean 1.87) can, in our view, be traced to a positive training effect. These observations concur with the results of the study by Sloan [20].

Furthermore, it has been shown that female students achieve significantly higher scores on the school-leaving exam (Abitur), although the range of scores for the sample in this study is severely limited as a result of its use as a criterion for university admission. Similar results are seen in an analysis of the data for over 126,000 students who passed the school-leaving exam thereby attaining the formal qualification for university study [http://www.it.nrw.de cited 2015 October 22]. However, it must be noted that the score on the school-leaving exam does not show any correlation with OSPE scores or those for the practical section of the state exam.

In comparison, for male students the score on the Physikum was higher and a correlation was seen between OSPE score and Physikum score showing that a stronger performance on the OSPE is accompanied by a stronger performance on the Physikum. This correlation could not be found in female students. No comparable studies have been published that could be cited in connection with these results. The fact is that, in contrast to the OSPE score which measures practical skill, the Physikum score involves a conglomerate of four oral assessments (anatomy, physiology, biochemistry, and prothodontics). The grade in prosthodontics includes a practical evaluation that could not be retrospectively filtered out in our study setting. For this reason it is not possible to identify which common factor or factors influence these two scores achieved by male students. Moreover, the analyses in this study revealed a correlation between study length and performance on the practical exam. A longer period of study correlates significantly with a lower exam score. Identical results were confirmed by a study in the field of dental education by Eberhard [21]. A longer length of study could compromise student motivation, or the sense of belonging to a particular semester cohort could suffer so much that any positive effects connected with belonging could be lost. In addition, the longer period of study could be an indication of external influences that affect not only the final grade but also the length of time spent studying at the university. It is conceivable that a student’s financial circumstances or family situation could assert this kind of influence. It would make sense that the influence is only present in female students if the sample population is classified by sex. It has already been shown that female students are more affected by multiple burdens such as pursuing academic study and meeting family responsibilities [22]. There are several indications in the results seen here—the low and moderately high correlations and small effect sizes—that other influential factors are missing from the model. This is not surprising since this study only takes performance measures and similar variables into account. Comprehensive personal profiles have not been available to date. Also, the data regarding the characteristics of the (testing) situation are incomplete. The reasons for these two limitations are found in the retrospective collection of the data. Another aspect which must be viewed critically is the fact that this study investigated only complete cases which can limit the validity of conclusions drawn from statistical analyses.

## 5. Conclusions

In light of the results seen here, it is possible to assert that the testing format of the OSPE partially predicts success on the practical section of the state exam covering conservative dentistry even if the results show, as expected, additional influences, such as length of study, and indicate the possible presence of other factors not visible in the data at hand. In future research, it would be worth the effort to monitor such factors over the course of study.

## Competing interests

The authors declare that they have no competing interests.

## Figures and Tables

**Table 1:**
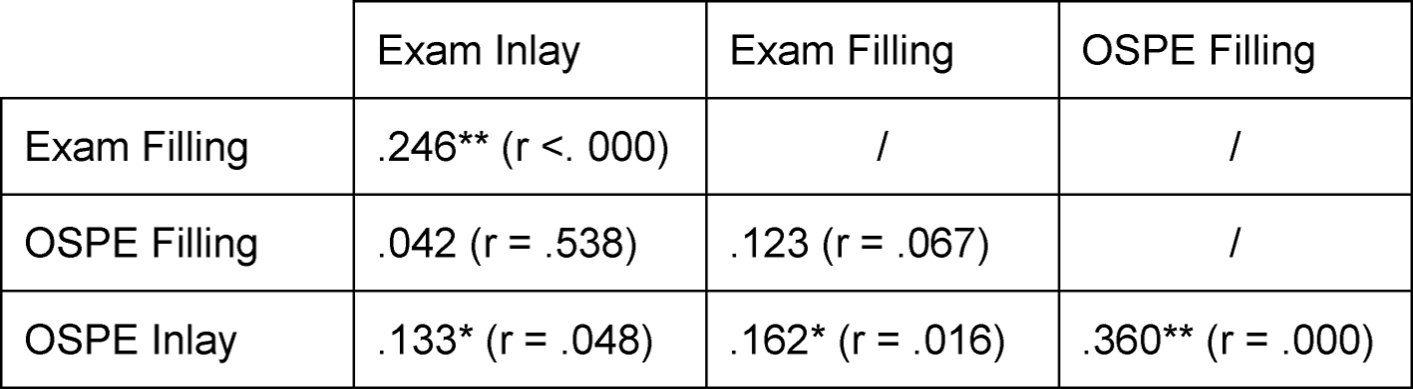
Correlation of the total and partial scores for the filling and inlay stations in both test settings (.**. The correlation is significant on the level of .01 (two-sided).
